# Doxorubicin-Induced Fetal Mesangial Cell Death Occurs Independently of TRPC6 Channel Upregulation but Involves Mitochondrial Generation of Reactive Oxygen Species

**DOI:** 10.3390/ijms22147589

**Published:** 2021-07-15

**Authors:** Anberitha T. Matthews, Hitesh Soni, Katherine E. Robinson-Freeman, Theresa A. John, Randal K. Buddington, Adebowale Adebiyi

**Affiliations:** 1 Department of Physiology, College of Medicine, University of Tennessee Health Science Center, Memphis, TN 38163, USA; anberitha@gmail.com (A.T.M.); drhiteshsoni@gmail.com (H.S.); krobin39@uthsc.edu (K.E.R.-F.); theresaadebola@yahoo.com (T.A.J.); 2 School of Health Studies, University of Memphis, Memphis, TN 38152, USA; rkb.btf@gmail.com

**Keywords:** doxorubicin, TRPC, Ca^2+^, glomerular mesangial cell, apoptosis, mitochondrial reactive oxygen species

## Abstract

Doxorubicin (DOX), a category D pregnancy drug, is a chemotherapeutic agent that has been shown in animal studies to induce fetal toxicity, including renal abnormalities. Upregulation of the transient receptor potential cation (TRPC) 6 channel is involved in DOX-induced podocyte apoptosis. We have previously reported that TRPC6-mediated Ca^2+^ signaling promotes neonatal glomerular mesangial cell (GMC) death. However, it is unknown whether DOX alters mesangial TRPC expression or viability in the fetus. In this study, cell growth was tracked in control and DOX-treated primary GMCs derived from fetal pigs. Live-cell imaging demonstrated that exposure to DOX inhibited the proliferation of fetal pig GMCs and induced cell death. DOX did not alter the TRPC3 expression levels. By contrast, TRPC6 protein expression in the cells was markedly reduced by DOX. DOX treatment also attenuated the TRPC6-mediated intracellular Ca^2+^ elevation. DOX stimulated mitochondrial reactive oxygen species (mtROS) generation and mitophagy by the GMCs. The DOX-induced mtROS generation and apoptosis were reversed by the mitochondria-targeted antioxidant mitoquinone. These data suggest that DOX-induced fetal pig GMC apoptosis is independent of TRPC6 channel upregulation but requires mtROS production. The mtROS-dependent GMC death may contribute to DOX-induced fetal nephrotoxicity when administered prenatally.

## 1. Introduction

Kidney cells, including mesangial cells, parietal epithelial cells, endothelial cells, and podocytes, sustain the structure and function of the glomerulus. Dysregulation of the associated cell functions is of pathological significance in a wide variety of diseases [1]. The central stalk of the glomerulus contains mesangial cells that line the inter-capillary space (mesangium) and generate extracellular matrix proteins [2,3]. The glomerular mesangial cells (GMCs) produce vasoactive agents and express G-protein-coupled receptors (GPCRs) and ion channels, including the transient receptor potential cation (TRPC) channels [2,3,4,5]. Activation of GPCRs and ion channels can contract or relax cultured GMCs to regulate their planar surface area, the physiological significance of which is unresolved in intact kidneys [2,3].

GMCs play critical roles in glomerulogenesis. Similar to endothelial cells, GMCs are recruited into the vascular clefts of developing glomeruli, where they organize the glomerular capillary network [6,7]. Formation of the capillary tuft and the commencement of filtration requires mesangial factors [6,7]. Thus, mesangiolysis and targeted deletion of critical mesangial cell genes, including ephrinB2, Notch, GATA3, and EBF1, have been demonstrated to alter mesangial and capillary loop maturation and impair glomerulogenesis [6,7,8,9,10,11].

Postnatal exposure to nephrotoxic medications, including aminoglycoside antibiotics and nonsteroidal anti-inflammatory drugs, can have short- and long-term adverse effects on immature kidneys and are a significant cause of acute kidney injury (AKI) and chronic kidney disease (CKD) [12,13]. Furthermore, since nephrogenesis ends by the 36th week of gestation in humans, medications administered to pregnant women or premature babies before completing nephrogenesis may alter kidney development and cause morphological and functional derangements of the nephrons [14,15]. Drug-induced impairment of kidney development may have long-term adverse consequences to kidney and cardiovascular functions.

The anthracycline antibiotic, doxorubicin (DOX), is a potent chemotherapeutic drug used to treat various cancers, including Hodgkin and non-Hodgkin lymphoma, and bone, breast, and liver, and ovarian cancers [16,17]. DOX promotes cardiac fibrosis and ventricular failure [16,17,18,19]. DOX treatment can also induce kidney injury and is an established rodent model of CKD [20]. DOX nephrotoxicity is characterized by damage to the glomerular capillaries, proteinuria, tubulointerstitial inflammation, and podocyte effacement [20]. DOX is a pregnancy category D drug as animal studies have shown evidence of toxic cardiac and kidney effects from its in utero exposure [21,22]. Administration of DOX to female rats four weeks before fertilization resulted in the fetuses exhibiting mesangial matrix accumulation, glomerulosclerosis, thickening of the glomerular basement membranes, and tubular injury [23,24]. Fetuses of rats that received DOX early in gestation have also been reported to exhibit hydronephrosis, cortical and medullary atrophy, and kidney lesions [21,25,26].

Normal proliferation, differentiation, and survival of kidney cells are critical processes during nephrogenesis [27,28]. Since an increase in cell growth or death can result in glomerular injury [29,30], mechanisms that control propagation and senescence are vital. This includes signal transduction pathways that can be modulated by changes in intracellular Ca^2+^ concentrations ([Ca^2+^]_i_) as [Ca^2+^]_i_ is a regulator of signal transduction processes controlling the cell cycle and survival [31,32]. Upregulation of TRPC6 channel expression has been demonstrated to contribute to DOX-induced podocyte apoptosis and glomerulosclerosis [33,34]. We have reported that TRPC6-mediated Ca^2+^ signaling promotes neonatal GMC death [35]. Whether DOX alters TRPC6 expression or mesangial viability in fetal GMCs is unclear. In this study, we examined the effects of DOX on fetal pig primary GMCs. We tested the hypothesis that DOX-induced upregulation of TRPC6 expression and TRPC6-dependent [Ca^2+^]_i_ elevation is associated with fetal GMC apoptosis.

## 2. Results

### 2.1. DOX Inhibited Fetal GMC Proliferation

**Figure 1:**
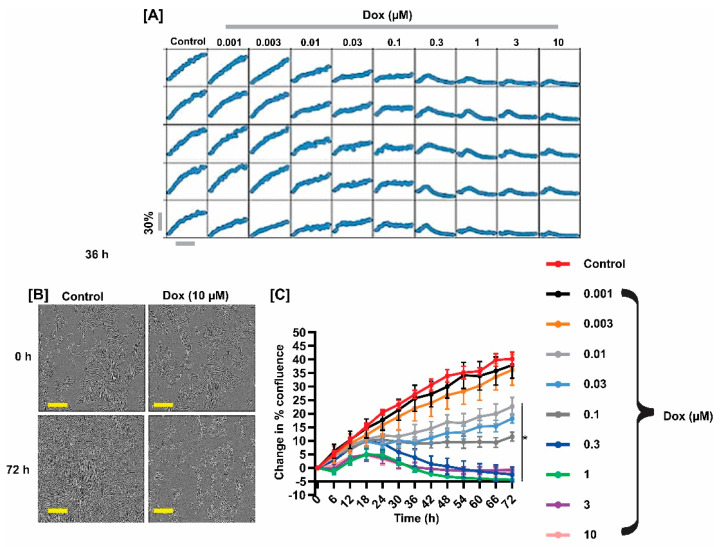
DOX inhibits fetal GMC proliferation. (**A**) Representative microplate graphs; (**B**) phase-contrast images; and (**C**) cell growth curves showing time- and concentration-dependent effect of DOX on fetal pig GMCs. Both control and DOX-treated cells show normal morphology at 0 h. DOX concentrations of 1 and 3 nM did not alter cell growth. Whereas 10–100 nM of DOX inhibited proliferation and 0.3–10 µM caused the death of the cells less than 30 h characterized by circular rather than elongated appearance. * *p* < 0.05 vs. control (10 nM: 30–72 h; 30 nM: 24–72 h; 100 nM: 30–72 h; 300 nM: 30–72 h; 1 µM: 24–72 h; 3 µM: 24–72 h; 10 µM: 24–72 h) (two-way ANOVA, with Tukey’s post hoc test) (*n* = 5 each). Scale bar = 300 µM.

Figure 1 shows the concentration- and time-response effects of DOX on fetal GMC proliferation. Automatic quantification of proliferation over 72 h indicated that 1 and 3 nM of DOX did not alter cell growth. Although 10–100 nM of DOX inhibited proliferation, 0.3–10 µM caused the death of the cells less than 30 h after treatment. These data suggest that DOX impedes proliferation and induces the death of fetal pig GMCs.

### 2.2. DOX Reduced TRPC6 Channel Expression in Fetal GMCs

**Figure 2:**
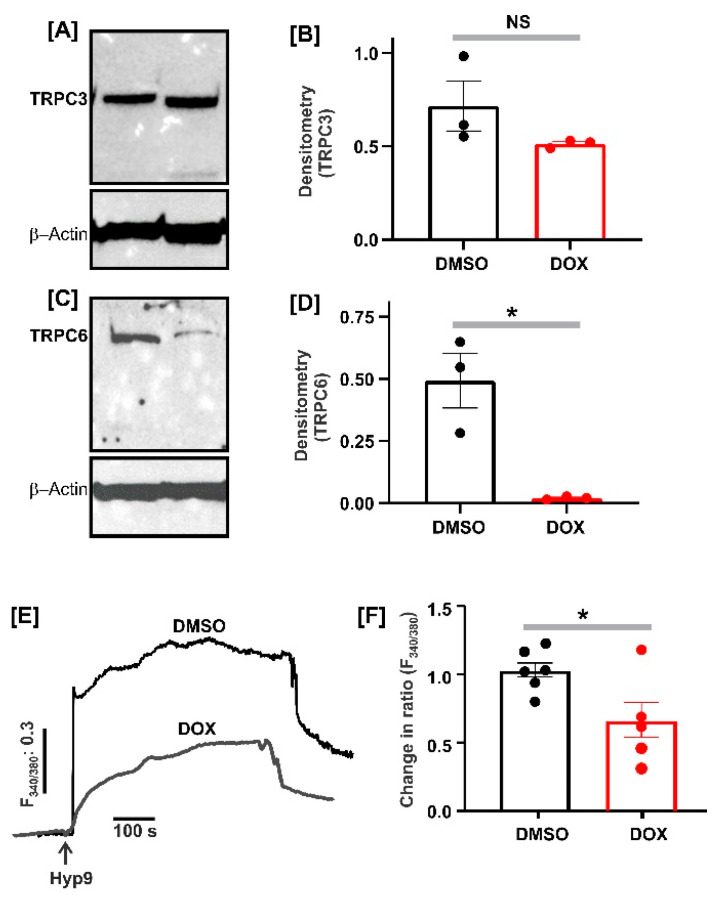
DOX reduces TRPC6 channel expression and TRPC6-mediated [Ca^2+^]_i_ in fetal GMCs. Western blot images and bar charts showing the expression levels of TRPC3 (**A**,**B**) and TRPC6 (**C**,**D**) in control (DMSO)- and DOX (100 nM)-treated fetal pig GMCs (18 h). (**E**,**F**) Exemplar traces and bar charts demonstrating the levels of Hyp9 (10 µM) (TRPC6 agonist)-induced [Ca^2+^]_i_ elevation in control (DMSO)- and DOX (100 nM)-treated fetal GMCs (18 h). * *p* < 0.05 vs. control (two-tailed unpaired *t*-test); NS = not significant.

Western immunoblotting of protein lysates isolated from fetal pig GMCs revealed TRPC3 and TRPC6 expression in the cells (Figure 2A–D). Although TRPC3 expression was not altered, the protein expression levels of TRPC6 were significantly reduced in GMC treated for 18 h with 100 nM of DOX (Figure 2A–D).

### 2.3. DOX Inhibited TRPC6-Mediated [Ca^2+^]_i_ Elevation in Fetal GMCs

TRPC6 channels regulate neonatal pig GMC [Ca^2+^]_i_ concentrations [35]. To determine whether DOX modulates TRPC6-dependent [Ca^2+^]_i_ elevation, we explored the effect of Hyp9, a TRPC6 channel activator on [Ca^2+^]_i_ levels. Hyp9 increased [Ca^2+^]_i_ in control fetal pig GMCs (treated with DMSO) (Figure 2E,F). However, DOX treatment attenuated the Hyp9-induced [Ca^2+^]_i_ elevation by ~36%.

### 2.4. DOX Stimulated Mitochondrial ROS Generation in Fetal GMCs

**Figure 3:**
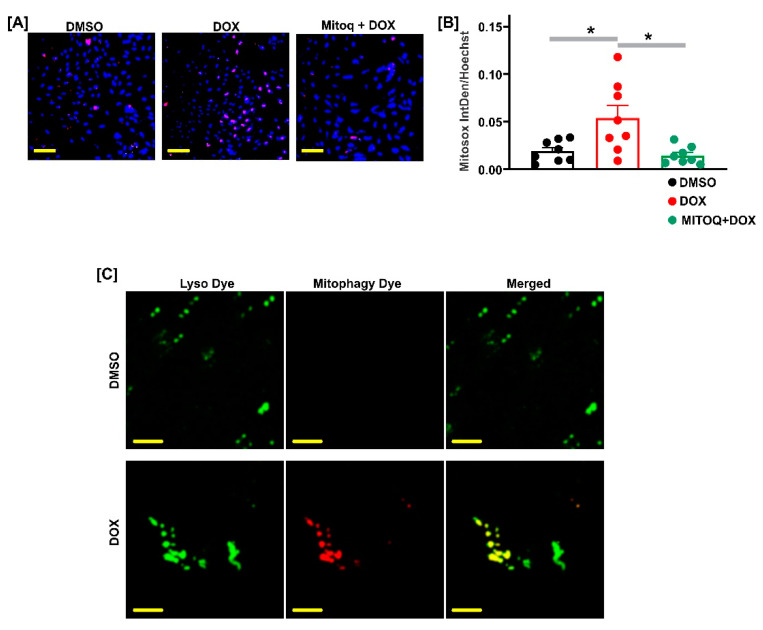
DOX stimulates mtROS generation in fetal GMCs. (**A**,**B**) Confocal microscopy images and charts showing increased MitoSOX Red staining (indicating ROS generation) in DOX (100 nM; 30 min)-treated cells and reversal by the mitochondria-targeted antioxidant mitoquinone (MitoQ 1 µM). (**C**) Representative confocal microscopy images (*n* = 4) showing induction of the mitophagy dye staining in DOX-treated cells. The mitophagy dye exhibits a weak basal fluorescence, but the fluorescence is induced when injured mitochondria fuse to the lysosome. The Lyso dye detects lysosomes in the cells. * *p* < 0.05 vs. DMSO/DOX (one-way ANOVA, with Tukey post hoc test). Scale bar = 100 µM.

DOX localizes to the mitochondria (mt) and promotes mt-dependent intracellular ROS generation in various cells. Here, we used the MitoSOX Red fluorogenic dye to evaluate superoxide generation, specifically in the mitochondria of live fetal pig GMCs. Oxidation from MitoSOX Red by superoxide produces red fluorescence, which was amplified in DOX-treated cells (Figure 3A,B). Pretreatment of the cells with the mitochondria-targeted antioxidant mitoquinone (MitoQ) decreased DOX-induced MitoSOX oxidation (Figure 3A,B). Increased mtROS stimulates mitophagy [36,37,38]; Figure 3C shows that mitophagy was essentially absent in the DMSO-treated fetal pig GMCs but was induced in the DOX-treated cells. Together, these data indicate that DOX stimulates mtROS generation in fetal GMCs.

### 2.5. MitoQ Reversed DOX-Induced Apoptosis in Fetal GMCs

**Figure 4:**
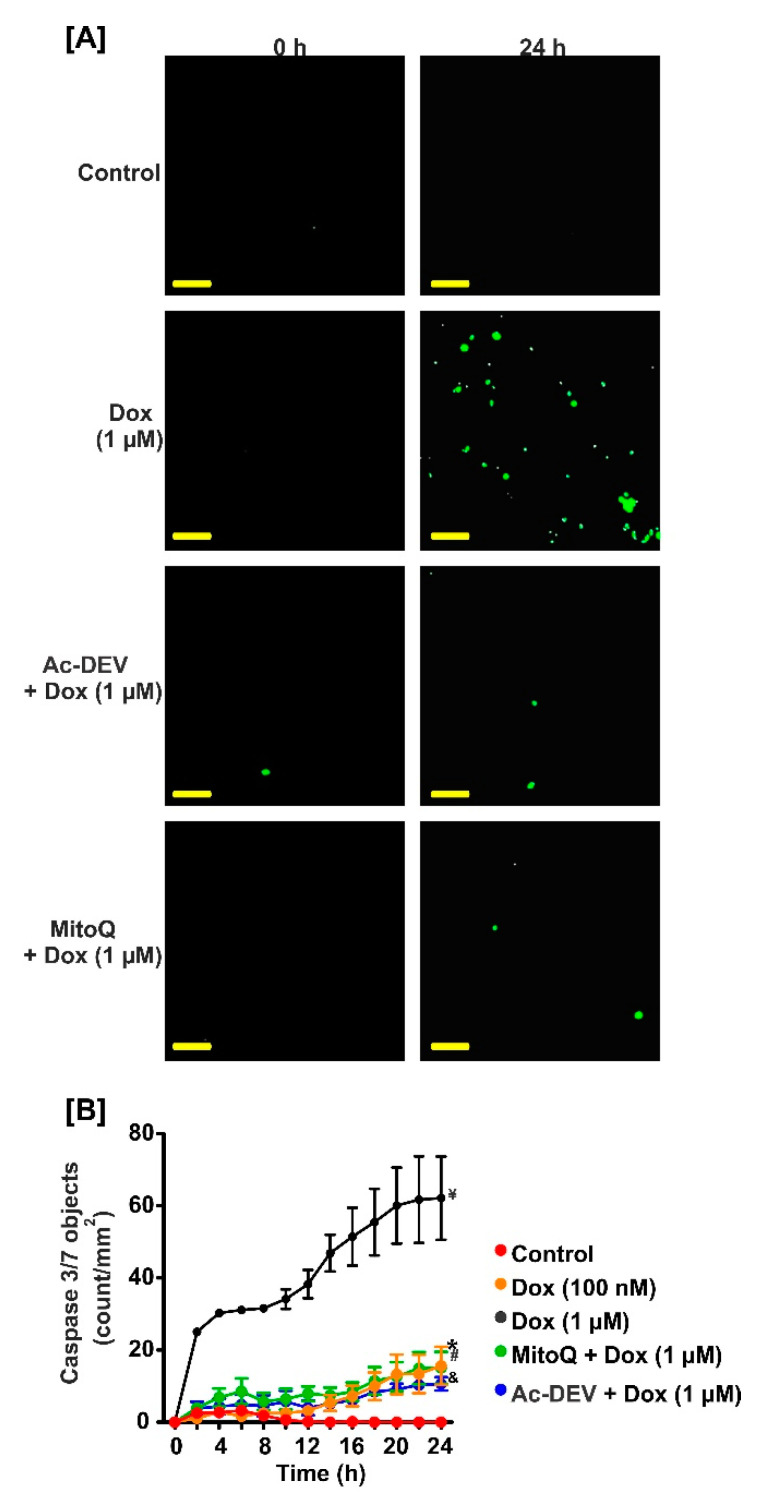
DOX-induced mtROS promotes caspase-3/7 activation in fetal GMCs. (**A**) Images (green fluorescence staining of the nuclear DNA in apoptotic cells) and (**B**) caspase 3/7 object count demonstrating that DOX induces apoptosis in fetal GMCs and reversal by MitoQ (1 µM) and Ac-DEVD-CHO (50 µM; a caspase-3 and caspase-7 inhibitor). ^¥^ *p* < 0.05 vs. control (2–24 h); * *p* < 0.05 vs. control (24 h) ^&,#^ *p* < 0.05 vs. DOX (1 µM; 2–24 h) (two-way ANOVA, with Tukey’s post hoc test); *n* = 5 each. Scale bar = 100 µM.

Figure 1 indicates significant cell death in the DOX-treated cells. To examine whether DOX induces fetal pig GMC apoptosis, we measured caspase-3/7 activity in the cells. As shown in Figure 4A,B, DOX engendered an increase in caspase-3/7 activity in a concentration- and time-dependent manner. Pretreatment of the cells with MitoQ and Ac-DEVD-CHO (a caspase-3 and caspase-7 inhibitor) reversed DOX-induced caspase-3/7 activation, indicating that mtROS mediates DOX-induced apoptosis in fetal pig GMCs (Figure 4A,B).

## 3. Discussion

The data presented here show that DOX inhibits the proliferation of fetal GMCs. The small and rounded appearance of the cells treated with ≥0.3 µM of DOX is indicative of cell death. DOX-induced apoptosis was confirmed by a concentration- and time-dependent increase in caspase-3/7 activity in the cells. The apoptotic activity of DOX is consistent with its effects on rat mesangial cells and other kidney cell types, including tubular and glomerular endothelial cells, and podocytes [39,40,41,42].

DOX kills cancer cells by inducing double-strand DNA breaks via intercalation into DNA and inhibiting topoisomerase-II-mediated DNA repair [16,43]. DOX also promotes cellular injury by generating deleterious ROS leading to oxidative DNA damage and cell death [16,43]. Increased renal TRPC6 expression is associated with podocyte injury and death in DOX nephropathy [33,44,45]. Treatment of mouse podocytes with DOX caused time-dependent apoptosis and was correlated with an increase in mRNA and protein expression of the TRPC6 channels [34]. Moreover, siRNA-mediated knockdown of TRPC6 reduced DOX-induced apoptosis in cultured mouse podocytes [34]. Together, these studies suggest that TRPC6-dependent Ca^2+^ signaling contributes to DOX-induced podocyte dysfunction.

TRPC6 is a member of the TRPC3/6/7 subgroup of cation channels within the TRPC family. These Ca^2+^ permeable channels share approximately 75% amino acid identity, are gated by diacylglycerol analogs, and co-assemble, forming a functional channel [46,47]. We have previously shown that TRPC6 activation and successive [Ca^2+^]_i_ elevation caused apoptosis in primary neonatal pig GMCs [35]. TRPC6-mediated GMC apoptosis was independent of ROS generation but involved induction of the calcineurin/NFAT, FasL/Fas, and caspase signaling pathways [35]. As a first step in determining whether DOX-induced upregulation of TRPC3 or TRPC6 is involved in fetal GMC apoptosis, we investigated the protein expression levels of these channels in DOX-treated cells. DOX did not change TRPC3 but reduced the protein expression levels of TRPC6 in fetal GMCs. Correspondingly, the TRPC6-mediated increase in [Ca^2+^]_i_ was significantly reduced in cells treated with DOX. These findings indicate that, unlike the podocytes, DOX does not promote TRPC6 upregulation in fetal pig GMCs. Instead, it reduced the expression of the channels. Hence, the TRPC6 channels upregulation may not contribute to fetal pig GMC death. The pathophysiological significance of DOX-induced reduction in TRPC6 protein expression levels requires further investigation.

Anticancer drugs, including DOX, are associated with cell death triggered by mitochondrial-dependent and -independent ROS production [48,49,50]. Oxidative stress-induced alterations in mitochondrial bioenergetics, loss of mitochondrial membrane potential, and disruption to the electron transport chain are mechanisms that underlie DOX-induced cellular dysfunction, especially in the cardiomyocytes [16,43,51,52]. However, the role of ROS in DOX-induced fetal mesangial cell death was unclear. We showed here that DOX engenders mtROS generation in fetal pig GMCs, an effect attenuated by the mitochondria-targeted antioxidant MitoQ. Mitophagy, a selective form of the autophagy mechanism that eliminates injured mitochondria, has been implicated in DOX cardiomyopathy [53,54,55,56]. Increased production of mtROS stimulates mitophagy [36,37,38]. In cardiac cells, DOX produced excessive elimination of the mitochondria via mitophagy [53,54,55,56]. Hence, our data showing that DOX triggered mitophagy in fetal pig GMCs supports the concept that DOX induces mtROS generation in the cells and promotes mitochondrial degradation. Furthermore, the reversal of DOX-induced apoptosis by MitoQ indicates that mitochondrial-derived oxidative stress is involved in DOX-induced fetal GMC apoptosis. Hence, pharmacological inhibition of mtROS could be a potential therapy for the treatment of DOX-induced fetal nephrotoxicity.

In summary, we demonstrated that DOX-induced fetal mesangial cell death occurs independently of TRPC6 channel upregulation but involves mtROS production. Further studies that use whole animal models are necessary to elucidate whether DOX-induced GMC death may contribute to its fetal nephrotoxic effects when administered prenatally.

## 4. Materials and Methods

### 4.1. Animals

Kidneys were harvested from fetuses delivered by caesarian section at 100–105 days of gestation (87–91% of term) from timed pregnancy sows of the same genetic lineage.

### 4.2. Primary GMC Culture

The fetal pigs were euthanized after delivery by euthasol (1 mL/kg; IV) followed by exsanguination (severing the abdominal aorta). After euthanasia, the kidneys were removed and placed in Dulbecco’s modified Eagle’s medium (DMEM; Life Technologies, Grand Island, NY, USA). Renal glomeruli were isolated from the fetal pigs by serial sieving of renal cortical homogenates using sterile stainless steel meshes. The glomeruli were decapsulated and cultured under conditions that favored GMC growth, as previously described [35,57].

### 4.3. Live-Cell Imaging

Real-time cell proliferation and kinetic quantification of apoptosis in fetal pig GMCs were performed using the IncuCyte ZOOM live content microscopy system (Essen Instruments, Ann Arbor, MI, USA) that has been previously described [35,57,58,59,60]. Briefly, GMCs were seeded in flat-bottom tissue culture plates and starved overnight by culturing in FBS/DMEM. The cells were treated with respective reagents, and the IncuCyte interface and software monitored their growth and kinetic activation of caspase-3/7.

### 4.4. Western Immunoblotting

Cultured GMCs were scrapped from flasks and homogenized in ice-cold RIPA buffer supplemented with a protease inhibitor cocktail (Thermo Scientific, Rockford, IL, USA). The proteins were then isolated and separated by 4–20% ExpressPlus PAGE Gels (GenScript, Piscataway, NJ, USA) and transferred onto PVDF membranes using a Semi-Dry Blotter (Thermo Scientific). The membranes were blocked with a 5% BSA blocking buffer for ~1 h at room temperature. The membranes were then probed overnight at 4 °C with respective primary antibodies. After a wash in Tris-buffered saline supplemented with 0.05% Tween 20 (TBST), the membranes were probed with horseradish peroxidase-conjugated secondary antibodies for 45 min at room temperature and washed in TBST. The membranes were then incubated with a chemiluminescence reagent (Thermo Scientific), and the immunoreactive protein bands were visualized and documented using the ChemiDoc imaging system (Bio-Rad Laboratories, Inc., Hercules, CA, USA).

### 4.5. Intracellular Ca^2+^ [Ca^2+^]_i_ Imaging

GMCs cultured in glass-bottom dishes were washed with PBS and incubated with Fura-2-acetoxymethyl ester (Fura-2 AM; 10 μM), and 0.5% pluronic F-127 for ~1 h at room temperature in modified Krebs’ solution (134 mM NaCl, 6 mM KCl, 1.2 mM CaCl_2_, 1 mM MgCl_2_, 10 mM HEPES, and 5.5 mM glucose, pH 7.4). Ca^2+^ imaging was performed using a ratiometric fluorescence system (Ionoptix Corp., Milton, MA, USA) that has been previously described [35,57,61].

### 4.6. Determination of Mitochondria ROS and Mitophagy Assay

The production of superoxides by the mitochondria was determined in live GMCs using the MitoSOX Red mitochondrial superoxide indicator Kit (Thermo Scientific). Live GMCs were loaded with 5 µM of the MitoSOX reagent and Hoechst 33342 nuclear stain for 10 min at 37 °C. Following 3 washes, the cells were immediately visualized, and random fluorescence images were documented using a Zeiss LSM 710 laser-scanning confocal microscope.

Mitophagy was documented in sparsely seeded GMCs using a mitophagy detection kit (Dojindo Molecular Technologies Inc., Rockville, MD, USA) following the manufacturer’s instructions. Briefly, the cells were washed with PBS and loaded with 100 nM of Mtphagy dye (mitophagy staining) for 30 min at 37 °C. The cells were then washed and treated with DMSO (control) or DOX for 18 h. The culture medium was removed, and cells were incubated in the dark with 1 µM Lyso dye (lysosome staining) at 37 °C for 30 min. The cells were washed with PBS, after which the co-localization between Mtphagy (Ex. 561 nM/Em. 650 nM) and Lyso (Ex. 488 nM/Em. 502–554 nM) dyes were documented with a Zeiss LSM 710 laser-scanning confocal microscope. 

### 4.7. Antibodies (Catalog Numbers Are in Parentheses) and Chemicals

Rabbit polyclonal anti-TRPC3 (AG1456), and anti-TRPC6 (ACC-120) antibodies were purchased from Abgent Inc. (San Diego, CA, USA) and Alomone Labs (Jerusalem, Israel), respectively. Mouse monoclonal anti-actin (ab3280) was purchased from Abcam (Cambridge, MA, USA). HRP-conjugated anti-rabbit (ab96919) and anti-mouse (ab98795) secondary antibodies were purchased from Abcam. All chemicals, unless otherwise stated, were purchased from Sigma-Aldrich (St. Louis, MO, USA). DOX and mitoquinone were purchased from LC Laboratories (Woburn, MA, USA) and MedKoo Biosciences (Morrisville, NC, USA), respectively.

### 4.8. Data Analysis

The Prism software (Graph Pad, Sacramento, CA, USA) was used for data analysis. Statistical significance was determined using the Student’s *t*-tests for unpaired data and the Tukey’s test for the analysis of variance for multiple comparisons. All data were expressed as the mean ± standard error of the mean (SEM). A *p*-value of <0.05 was considered significant.

## Data Availability

The data presented in this study are available upon reasonable request from the corresponding author.
